# A Rare Case of Fulminant Myocarditis Caused by COVID-19 and Influenza B Co-infection

**DOI:** 10.7759/cureus.39905

**Published:** 2023-06-03

**Authors:** Noreen Mirza, Mariam Mirza, Mohammad Nabil Rayad, Zaid Ahmad Amin, Addi Suleiman

**Affiliations:** 1 Internal Medicine, Saint Michael’s Medical Center, Newark, USA; 2 Internal Medicine, St. George’s University School of Medicine, True Blue, GRD; 3 Cardiology, Saint Michael’s Medical Center, Newark, USA

**Keywords:** covid-19, pocus in critical care and mechanical circulatory support (vv and va ecmo), iabp, intra-aortic balloon pump (iabp), influenza virus type a and b, viral-induced myocarditis, influenza b, covid-induced myocarditis, fulminant myocarditis

## Abstract

Coronavirus disease 2019 and influenza B can have similar presentations and are self-limited in most cases. They are rarely associated with fatal cardiovascular complications. Coronavirus and influenza B-induced myocarditis is a rare but reversible cause of cardiogenic shock. Early detection plus administration of antiviral agents and supportive care with mechanical circulatory support in the form of an intra-aortic balloon pump can be a lifesaving measure in myocarditis.

## Introduction

Coronavirus disease 2019 (COVID-19) caused by severe acute respiratory syndrome coronavirus 2 (SARS-CoV-2) has changed the face of medicine resulting in more than 230 million confirmed cases of the disease since December 2021 [1]. This disease has varying clinical presentations, with the most common organ involved being the lung [2]. Patients with underlying cardiovascular conditions are seen to have prognostic consequences [2,3]. Only a few cases of COVID-19-related myocarditis have been described in the literature [4-6]. Most of these cases are related to messenger RNA (mRNA) vaccinations in the younger population [7]. Influenza infection is highly transmissible between individuals [8]. It commonly presents with upper respiratory symptoms and is self-limiting in most cases; however, it may progress to acute respiratory distress syndrome and myocarditis [8]. Influenza A is more frequently associated with the development of myocarditis [9]. Myocarditis is an inflammatory disease of the heart that can manifest as cardiac arrhythmias, chest pain, and heart failure. Heart failure can progress to cardiocirculatory failure causing fulminant myocarditis [9]. Here, we present a rare case of a previously healthy male patient with COVID-19 and influenza B-induced fulminant myocarditis requiring an intra-aortic balloon pump (IABP).

## Case presentation

A 45-year-old male with an alcohol and tobacco use history presented to the emergency department after he was found confused and lying on the street. He complained of exertional shortness of breath without orthopnea or paroxysmal nocturnal dyspnea. He denied chest pain or presyncope. The patient was unvaccinated against COVID-19 or influenza. On examination, his temperature was 98.9°F, heart rate was 130 beats per minute, blood pressure was 144/111 mmHg, respiratory rate was 20 breaths per minute, and he was saturating 97% on room air. Additionally, there were bilateral bibasilar crackles in the lungs and trace lower extremity edema bilaterally. Initial laboratory data are shown below in Table 1.

**Table 1:** Initial laboratory data on the day of admission. BUN = blood urea nitrogen; AST = aspartate transaminase; ALT = alanine transaminase; CK = creatinine kinase; WBC = white blood cell; SARS-CoV-2 = severe acute respiratory syndrome coronavirus 2; NAA = nucleic acid amplification; RSV = respiratory syncytial virus

	Admission labs	Reference range
Sodium	138 mmol/L	136–145 mmol/L
Potassium	4.0 mmol/L	3.5–5.3 mmol/L
Bicarbonate	20 mmol/L	20–31 mmol/L
Chloride	103 mmol/L	98–110 mmol/L
Anion gap	11	<12
B-natriuretic peptide	187 pg/mL	0–100 pg/mL
High-sensitivity troponin	1,777 ng/L	<78 ng/L
BUN	44 mg/dL	6–24 mg/dL
Creatinine	3.7 mg/dL	0.6–1.2 mg/dL
Lactic acid	2.1 mmol/L	0–2.0 mmol/L
C-reactive protein	9.4 mg/dL	0.0–0.8 mg/dL
Procalcitonin	5.05 ng/mL	<0.5 ng/mL
Total bilirubin	2.1 mg/dL	0.2–1.2 mg/dL
AST	128 U/L	10–36 U/L
ALT	59 U/L	9–46 U/L
Alkaline phosphatase	100 U/L	40–115 U/L
Total CK	288 U/L	38–176 U/L
Complete blood count		
WBC count	14,400 cells/µL	4,400–11,000 cells/µL
Hemoglobin	10.1 g/dL	13.5–17.5 g/dL
Platelet count	140,000 cells/µL	150,000–450,000 cells/µL
SARS antigen	Positive	Negative
SARS-CoV-2 by NAA	Positive	Negative
Rapid influenza B Ag	Positive	Negative
RSV screen	Negative	Negative

**Figure 1:**
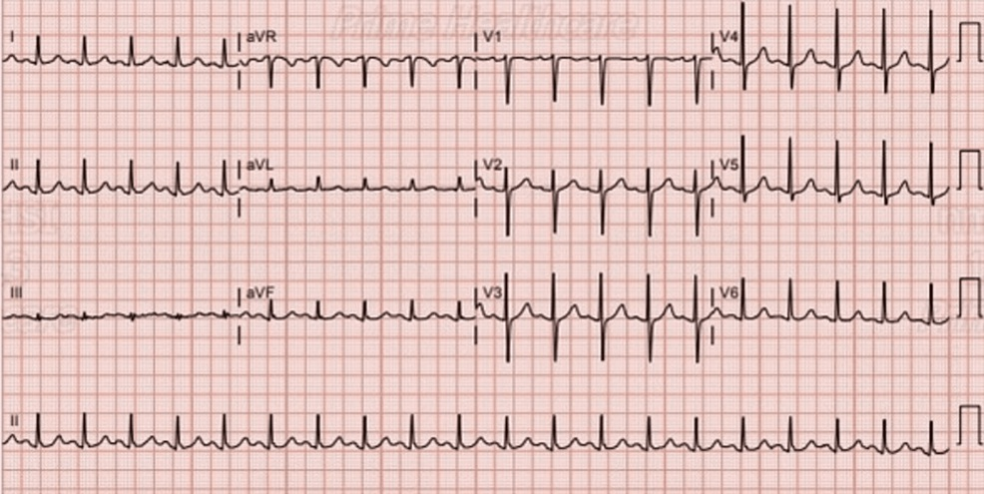
Electrocardiogram on the day of admission showing sinus tachycardia with no ST or T-wave changes.

**Figure 2:**
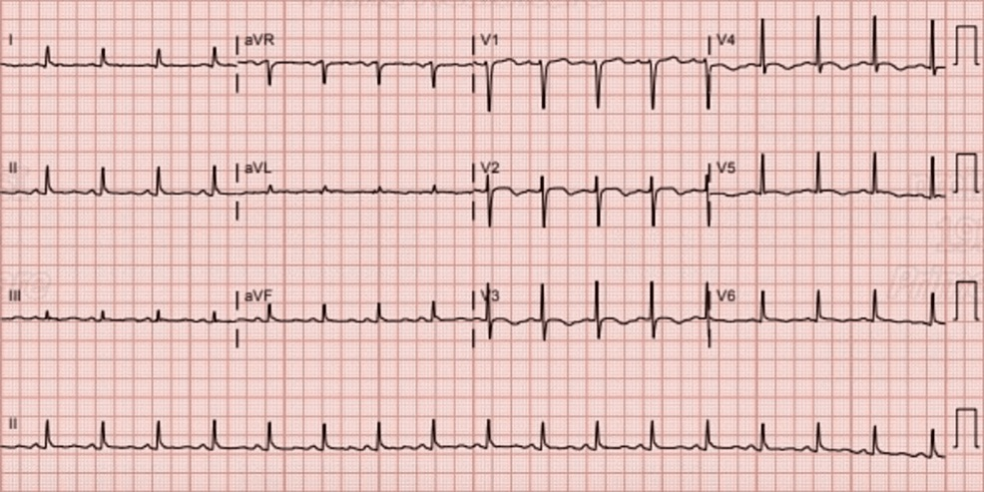
Electrocardiogram 16 hours after admission showing sinus rhythm with T-wave inversions in the anterolateral leads.

An electrocardiogram (ECG) on admission showed sinus tachycardia with a normal axis and no acute ST-T-wave changes (seen in Figure 1); however, a subsequent ECG 16 hours later showed sinus rhythm with T-wave inversions (seen in Figure 2).

Echocardiogram showed a severely reduced left ventricular ejection fraction (LVEF) of 30-35% with severe hypokinesis of the mid and apical left ventricle (LV), as seen in Videos 1-3.

**Video 1:** Apical four-chamber view.

**Video 2:** Parasternal short-axis view.

**Video 3:** Parasternal long-axis view.

**Figure 3:**
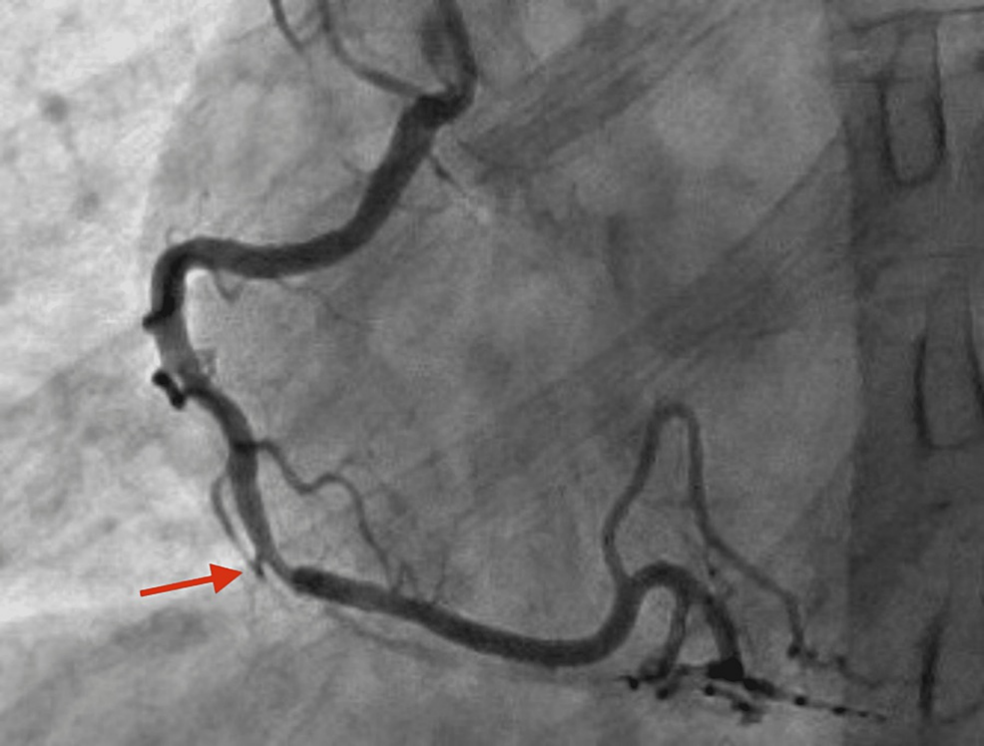
Coronary angiogram of the right coronary artery: large size, dominant vessel, and mildly calcific mid 60% lesion with a negative instantaneous wave-free ratio of 0.96.

**Figure 4:**
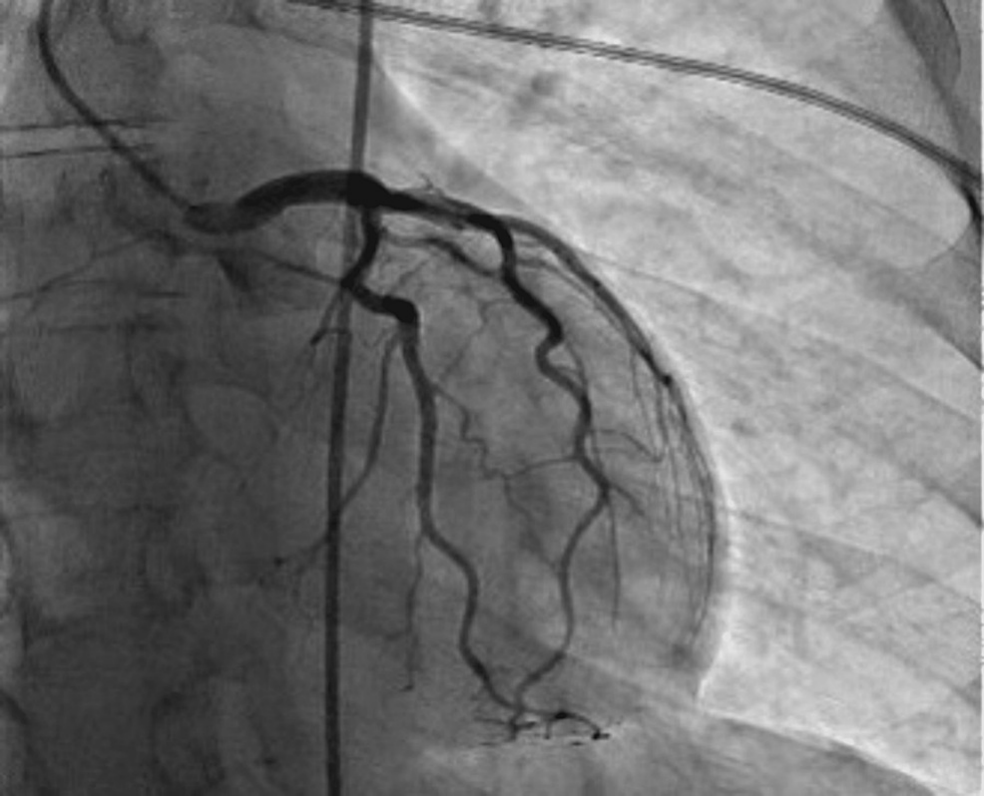
Coronary angiogram showing left anterior descending artery, left circumflex artery, and left main coronary artery with no significant stenosis.

The patient was empirically started on antibiotics with ceftriaxone and doxycycline. He was started on antiviral therapy with oseltamivir and was given remdesivir. After the patient developed ECG changes and his troponins continued trending upwards peaking at 1,876 ng/L, the decision was made to start acute coronary syndrome protocol with a therapeutic heparin drip, aspirin, Brilinta, and atorvastatin. The patient then underwent cardiac catheterization that showed non-obstructive 60% stenosis in the distal right coronary artery with negative physiologic testing, as seen in Figure 3. Cardiac catheterization was otherwise unremarkable in the other coronary arteries, as seen in Figure 4.

During the procedure, the patient’s blood pressure dropped requiring vasopressors. The patient underwent IABP insertion for hemodynamic support in light of findings of severe LV dysfunction with hypotension. The patient’s ejection fraction and hemodynamic status improved over a few days and the IABP was removed.

## Discussion

Myocarditis typically resolves on its own; however, some cases may progress to severe conditions, including myocardial infarction, heart failure, and arrhythmias [10]. Myocarditis in COVID-19 has been hypothesized to be related to a cytokine storm [10]. The virus is able to gain access to human cells by attaching its spike protein to the membrane protein called angiotensin-converting enzyme 2 (ACE2). The spike protein is then cleaved at the S1/S2 and S2 sites so binding to ACE2 may occur [11]. TMPRSS2, a serine protein, is responsible for the cleavage of the S1/S2 site. ACE2 is present in cardiomyocytes allowing SARS-CoV-2 to infect the human heart [10]. When the virus enters cardiomyocytes, its accessory proteins may impede stress granule formation allowing for viral replication. Antigen-presenting cells (APCs) can prime naive T-lymphocytes which travel to the cardiomyocytes and cause myocardial inflammation through cell-mediated cytotoxicity. During the cytokine storm syndrome, proinflammatory cytokines are released into the bloodstream which boosts T-lymphocyte activation, resulting in further cytokine production and causing a feedback loop of damage to the myocardium [10].

Influenza A is a more common cause of myocarditis, and the pathogenesis behind myocarditis in influenza is still unknown. It is hypothesized to be due to direct myocardial injury by the influenza virus and heightened levels of host cell immunity due to increased expression of matrix metalloproteinases, trypsin, and cytokines such as tumor necrosis factor [12]. The cytokines result in an overproduction of nitric oxide which leads to myocardial ATP depletion due to the inhibition of complexes I and II of the electron transport chain [12]. There is also an imbalance between levels of Bax versus anti-apoptotic protein B-cell lymphoma 2 leading to the induction of cardiomyocyte apoptosis [12].

Coronavirus and influenza viruses share common clinical manifestations, including respiratory symptoms, usually fever, cough, fatigue, and myalgia, and are typically spread through respiratory droplets or aerosols [13]. Co-infection of both viruses is a rare phenomenon and is typically seen with influenza A but not as often with influenza B [13]. The severity of clinical symptoms in the presence of a positive influenza test should raise the clinical suspicion of co-infection with COVID-19, as was seen in our patient [13]. In a UK-based study regarding co-infection of COVID-19 and influenza, it was found that viral co-infection was associated with higher rates of mortality and invasive mechanical ventilation [14]. Another study published in 2020 from China found that concurrent infection of both viruses caused patients to have higher risks of inferior health outcomes [15]. A limited number of cases have been published in the literature regarding co-infection, and the most common laboratory findings that have been seen in co- infection include leukopenia, elevated C-reactive protein, elevated levels of liver transaminases, and lymphopenia [13]. The most frequent complications of co-infection include acute respiratory distress syndrome and acute liver injury [13]. There has also been one report of stress-induced cardiomyopathy precipitated by COVID-19 infection and influenza A co-infection [16].

Myocarditis treatment is focused on both hemodynamic and respiratory support [17]. In terms of hemodynamic management, it begins with medication management with vasopressors and inotropes [17]. However, more severe cases of myocarditis may require the use of an IABP, extracorporeal membrane oxygenation, or ventricle assist device [17]. An IABP allows for circulatory support by increasing systolic blood pressure while reducing cardiac afterload and myocardial oxygen demands [17]. IABP used within the first 24 hours of presentation has a reduced risk of mortality from myocarditis [17]. Myocarditis can progress to fulminant acute heart failure and cardiogenic shock. Early treatment with IABP restores hemodynamic stability by improving LV function and reducing LV volume and pressure and overall improves survival [18]. IABP is associated with an increase in LVEF by about 8% which is an important determinant of survival [18].

## Conclusions

This case highlights a rare presentation of COVID-19 and influenza co-infection-induced fulminant myocarditis where patients can have improved LVEF within a few days with mechanical support devices such as IABP.
